# Early childhood caries and its relationship with perinatal, socioeconomic and nutritional risks: a cross-sectional study

**DOI:** 10.1186/1472-6831-14-47

**Published:** 2014-05-06

**Authors:** Valdeci Elias dos Santos Junior, Rebeca Maria Brasileiro de Sousa, Maria Cecília Oliveira, Arnaldo França de Caldas Junior, Aronita Rosenblatt

**Affiliations:** 1 Department of Paediatric Dentistry, Faculty of Dentistry, University of Pernambuco, Recife, Brazil; 2 Rua São Sebastião, 417 #101 CEP 54410500, Jaboatão dos Guararapes, PE, Brazil

**Keywords:** Dental caries, Child, Obesity, Teenage pregnancy, Birth weight, Prematurity

## Abstract

**Background:**

Socioeconomic, perinatal and other life cycle events can be important determinants of the health status of the individual and populations. This study aimed to assess the prevalence of early childhood caries (ECC), perinatal factors (gestational age, teenage pregnancy and birth weight), family income and nutritional risk in children.

**Methods:**

A cross-sectional study in which 320 children were examined according to the criteria established by the World Health Organization. A previously validated questionnaire was used to obtain information from parents and guardians about family income, gestational age and birth weight. To check the nutritional risk, we used the criteria provided by the CDC (Center for Disease Control). For Statistics, Pearson’s, chi-square and the multivariate Poisson analyses were used to determine the association among variables.

**Results:**

Approximately 20% of children had ECC, and the Poisson multivariate analyses indicated that family income (p = 0.009), birth weight (p < 0.001) and infant obesity (p < 0.001) were related to the increase of ECC, and gestational age was not significantly associated with ECC (p = 0.149). Pregnancy in adolescence was not included in the regression analyses model because it was not statistically significant in the chi-square test (p > 0.05).

**Conclusion:**

The prevalence of ECC was related to low family income, premature birth and infant obesity.

## Background

Early childhood caries is a common public health problem in developing countries, where malnutrition is still an issue. This pattern of decay develops very rapidly in preschoolers, affecting tooth surfaces that are naturally caries-prone [1,2]. Whereas the disease is influenced by social context, this discussion goes beyond the boundaries of oral health issues to social equality [3].

Adverse perinatal factors result in poor oral health conditions, considering that low birth weight children tend to develop enamel and dentine defects, which facilitates the adhesion and colonization of cariogenic bacteria in poorly calcified dental tissue [2,4]. The current literature [3-5] indicates that there is a strong association between enamel defects, prematurity and low birth weight [6].

According to The World Health Organization [7], adolescent pregnancy is a health risk that affects mothers and newborns that also increases disadvantageous social and biological life events [7]. Prematurity and low birth weight is closely related to infant mortality, perinatal infections and the poor growth and development of children [8].

Therefore, this study aimed to assess the prevalence of early childhood caries and its association with perinatal factors, such as gestational age, pregnancy and birth weight, along with family income and nutritional risk, to produce incremental evidence-based knowledge to update the state of care in a fast-growing society.

## Methods

This is an epidemiological study conducted in Cabo de Santo Agostinho, in the southeastern part of Pernambuco, Brazil, which has a population of approximately 185,123 inhabitants [9]. The sample was comprised of 320 preschoolers aged from three to four years who were attending municipal kindergarten.

The calculation of sample size considered that a previous pilot study on ECC indicated a prevalence of 22.3%, with a 95% confidence interval and a 5% standard error. This resulted in a sample of 266 children, and after adding 20% to avoid data loss, a sample size of 320 children was established.

The pilot study was conducted with 10% of the sample in the yards of the schools, in a knee-to-knee position, with natural light and using a dental mirror and wooden spatula. Children received oral hygiene instruction and supervised tooth brushing before the exam. The children who took part in this trial were included in the final sampling.

This study was approved by the Ethics Committee of the University of Pernambuco. (Protocol No. 119/12). Consent for undertaking the research was obtained from the school principals, and consent to perform the examinations came from the parents or guardians. Only the children of those parents or guardians who returned the signed permission forms were included in the study.

The examinations were performed by a calibrated examiner for visual exam following criteria established by the World Health Organization [10]. Kappa coefficients for intra-examiner agreement was K = 0.90.

Cavitated carious lesions in children younger than 71 months were classified as ECC, according by Drury et al. [11]. The individuals with syndromes that impact the oral cavity were excluded from the sample to avoid confounding factors.

In addition to the oral examination, children were measured and weighed to assess nutritional risk as proposed by the CDC (Center for Disease Control) [12], which analyses the BMI (Body Mass Index) curve by age and sex from 2–19 years of age. The child was considered to be underweight when the value was below the 5th percentile, normal weight when between the 5th (inclusive) and below the 85th, overweight when between the 85th (inclusive) and 95th and obese when above the 95th percentile. The measurement of body weight was recorded by the researcher, with the child standing with minimal clothing without shoes on a portable calibrated scale with a precision of 100 g, and the height was measured with a tape strip scale.

The examiners administered a questionnaire, which was previously validated in a pilot study, to parents and guardians at the pick-up time at school in order to obtain information on family income. To determine the gestational age and birth weight, we accessed the vaccination and the local maternity hospital records that followed the WHO criteria [13], indicating preterm to be less than 37 weeks. The WHO criteria [8] for adolescent pregnancy included girls from 10 to 19 years of age. The classification for birth weight included very low birth weight (less than 1500 g), low birth weight (less than 2500 g) and normal birth weight (equal to or greater than 2500 g).

After data collection and the categorization of variables, we created a database for statistical analysis using SPSS (Statistical Package for Social Sciences) version 17. To test the association between two categorical variables, the chi-squared test was used. To explain the prevalence of early childhood caries and its association with the other risk factors, we chose the Poisson regression test, which is similar to logistic regression, with the advantage that the prevalence ratio (PR) is more stable and the value for OR varies in a smaller range. The margin of error was 5%.

## Results

Three hundred and twenty preschoolers were evaluated. There were no losses or drop-outs because the schools were not very far from each other and we could always return to see those that failed to attend school regularly. The mean age was 43.2 months. The presence of ECC was registered as yes/no and results showed that 20% of children had this type of dental disease.

The prevalence of ECC was shown to be related to low family income, low birth weight, infant obesity and shorter gestational age (p < 0.05). There was a higher prevalence of ECC among children with low birth weight (80.4%) than those born with a normal weight (9.9%) and those born preterm (82.8%) compared with those born at term (13.7%) (Table 1). Twenty-five percent (80 cases) involved adolescent pregnancy. Table 2 shows the results for the multivariate Poisson regression analysis to explain the association of early childhood caries with the following variables: family income, birth weight, gestational age and nutritional risk. For significant variables, the probability of the presence of early childhood caries increased if the child was underweight at birth (p < 0.001), demonstrated infant obesity (p < 0.001) or exhibited a family income less than or equal to $ 282.00 (p = 0.009).

**Table 1:** Early childhood caries, family income, adolescent pregnancy, birth weight, gestational age and nutritional risk

	**Evaluation ECC**							
**Variable**	**YES**		**NO**		**TOTAL**		**P value**	**PR (IC a 95%)**
	**N**	**%**	**n**	**%**	**N**	**%**		
**TOTAL**	**64**	**20.0**	**256**	**80.0**	**320**	**100.0**		
• **Family income**								
No Income	14	34.1	27	65.9	41	100.0	p^(1)^ = 0.01*	3.34 (1.71 to 6.51)
Less than or equal to minimum wage ($ 282.00)	37	24.3	115	75.7	152	100.0		2.38 (1.32 to 4.27)
More than one minimum wage	13	10.2	114	89.8	127	100.0		1,00
• **Adolescent pregnancy**								
Yes	15	18.7	65	81.3	80	100,0	p^(1)^ >0.18	1.16 (0,63 to 2,01)
No	38	16	204	84	240	100,0		1,00
• **Birth weight**								
Low	37	80.4	9	19.6	46	100.0	p^(1)^ < 0.01*	8.16 (5,55 to 12.00)
Normal	27	9.9	247	90.1	274	100,0		1.00
• **Gestacional age**								
Premature	24	82.8	5	17.2	29	100.0	p^(1)^ < 0.01*	6.02 (4.32 to 8.39)
Term	40	13.7	251	86.3	291	100.0		1.00
• **Nutritional Risk**								
Underweight	2	100,0	-	-	2	10,0	p^(1)^ = 0.01*	**
Normal weight	57	18.4	253	81.6	310	100.0		
Obesity	5	62.5	3	37.5	8	100.0		

(*)Significant association at 5.0%.

(**)Unable to determine due to the occurrence of null frequencies.

(^1^) By chi-square test.

**Table 2:** Results of multivariate poisson regression to the prevalence of early childhood caries

	**PR and IC 95,0%**		
**Variables**	**Univariate**	**Adjusted**	**P value**
• **Family income**			
No	3.58 (1.80 a 7.12)	2.42 (1.20 a 4.88)	p = 0.009*
Less than or equal to minimum wage ($ 282.00)	2.50 (1.36 a 4.61)	2.15 (1,30 a 3.54)	
More than one minimum wage	1,00	1,00	
• **Birth weight**			
Low	8.40 (5.66 a 12.46)	7.09 (3.91 a 12.86)	p < 0.001*
Normal	1,00	1,00	
• **Gestational age**			
Premature	6.11 (4.35 a 8.58)	1.39 (0.89 a 2.19)	p = 0.149
Term	1.00	1.00	
• **Risk nutritional**			
Normal weight	1.00	1.00	p < 0.001*
Obesity	3,40 (1.89 a 6.11)	6,24 (3.06 a 12.72)	

(*):Significant at 5.0%.

Pregnancy in adolescence was not included in the statistical regression model because it did not show a statistically significant difference in the person's chi-squared test (p > 0.05).

## Discussion

The present study is in accordance with previous reports [14-16] that describe the strength of the relationship between decreases in family income, the risk of children developing ECC and the impact of this social determinant of health. Lagreca [17] conducted a survey that reported the incidence of dental caries in relation to the socioeconomic status that indicated that in Brazil, children whose family income was in the range of the minimum wage, caries incidence was 57% higher than those whose family income was above four times the minimum wage. Then, with the speed of the production and the diffusion of new and advanced science, it would be anachronistic to continue to refer to the aetiology of dental caries exclusively by the intersection of primary factors, such as microbiota and host substrate.

According to the Brazilian Institute of Geography and Statistics-IBGE [9], 53.9 million Brazilians live in extreme poverty, which accounts for 31.7% of the population. In the northeastern part of the country, 76.5% of the families earn less than the minimum wage, which is approximately that found in the present study: 60.3% of families living in poverty.

From the perspective of health inequality, Marmot, Bell and Goldblatt [18] conceptualized that there is a convergence between unfavourable socioeconomic conditions and the presence of diseases. Corroborating this concept, the present study indicated that children from families that earn less than $ 282 are at a higher risk for ECC.

It has been suggested that human life events can alter stable biological mechanisms and turn them into a genetic legacy process called incorporation of biologic features [19]. From this perspective, the traditional etiological factor for caries, which involves the association of high sugar intake [20,21], poor oral hygiene [22-24], lack of exposure to fluoride and perinatal disturbances related to enamel defects [21,24], which could influence genetic legacy.

Currently, reports [25,26] show that prematurely born individuals have poor mineralization of the teeth and poorer oral health indicators, supporting the evidence of the role played by enamel defects in the development of ECC. Moreover, in a 7-year cohort study, Targino et al. (2011) [24] revealed that enamel defects constitute a risk factor for the development of early childhood caries. Thus, perinatal factors such as low birth weight and gestational prematurity are risk factors for the development of early childhood caries.

Due to the worldwide increase in childhood obesity across populations and the polarization of dental caries, several studies [27,28] reported on the associations of obesity and infant caries; however, the results of these studies are still controversial. In 2006, a systematic review [29] found only one study that consistently showed a direct association between obesity and dental caries with a high level of evidence. The findings of this work show an association between childhood obesity and dental caries, as in some previous epidemiological and cohort studies [27,28]. The results of this study indicate that it is possible that the conflicting results could be related to variations in the way data were collected, the socioeconomic status of the sample, the parameters used to analyse nutritional status and caries diagnosis [29].

Although the present research indicated no significant association between adolescent pregnancy and ECC, perinatal complications arising from the lack of biological development of teenagers are themselves risk factors for preterm and low birth weight children [25], which were identified as risk factors for ECC [29].

## Conclusion

The prevalence of ECC was shown to be related to low family income, premature birth and infant obesity. Thus, this study showed that socio-economic factors and perinatal events are important determinants for the status of oral health in children.

### Bullet points

•It is essential to monitor the prevalence of early childhood caries and its risk factors.

•Low family income, premature birth and infant obesity should be considered as risk factors for the development of ECC.

## Competing interest

This study was funded by the Ministry of Education of Brazil. Thus, there is no conflict of interest, nor any such interference with the results of this article.

## Authors’ contributions

VSJ participated in the study design and epidemiological data analysis and drafted the manuscript. CMC facilitated the field work and data collection. RMB was the main dentist who examined and diagnosed all of the children. AR and AFCJ were the main supervisors, and they guided the study design, performed statistical analysis and drafted the manuscript. All authors read and approved the final manuscript.

## Pre-publication history

The pre-publication history for this paper can be accessed here:

http://www.biomedcentral.com/1472-6831/14/47/prepub
